# Genetic mapping of *AhVt1*, a novel genetic locus that confers the variegated testa color in cultivated peanut (*Arachis hypogaea* L.) and its utilization for marker-assisted selection

**DOI:** 10.3389/fpls.2023.1145098

**Published:** 2023-03-20

**Authors:** Hao Chen, Xinlei Yang, Rirong Xu, Xiangyu Chen, Haifeng Zhong, Nian Liu, Li Huang, Huaiyong Luo, Dongxin Huai, Wenjing Liu, Yuhua Chen, Jianhong Chen, Huifang Jiang

**Affiliations:** ^1^ Institute of Crop Sciences, Fujian Academy of Agricultural Sciences, Fujian Research Station of Crop Gene Resource and Germplasm Enhancement, Ministry of Agriculture and Rural Affairs of People’s Republic of China, Fujian Engineering Research Center for Characteristic Upland Crops Breeding, Fujian Engineering Laboratory of Crop Molecular Breeding, Fuzhou, China; ^2^ Oil Crops Research Institute of the Chinese Academy of Agricultural Sciences, Key Laboratory of Biology and Genetic Improvement of Oil Crops, Ministry of Agriculture and Rural Affairs of People’s Republic of China, Wuhan, China; ^3^ State Key Laboratory of North China Crop Improvement and Regulation, North China Key Laboratory for Crop Germplasm Resources of Education Ministry, Key Laboratory for Crop Germplasm Resources of Hebei, Hebei Agricultural University, Baoding, China; ^4^ Institute of Quality Standards and Testing Technology for Agro-Products, Fujian Academy of Agricultural Sciences, Fujian Key Laboratory of Agro-products Quality and Safety, Fuzhou, China; ^5^ R&D Center for Oil Crops, Quanzhou Institute of Agricultural Sciences, Jinjiang, China

**Keywords:** variegated testa color, BSA-seq, peanut (*Arachis hypogaea* L), genetic mapping, marker- assisted selection

## Abstract

**Introduction:**

Peanut (Arachis hypogaea L.) is an important cash crop worldwide. Compared with the ordinary peanut with pure pink testa, peanut with variegated testa color has attractive appearance and a higher market value. In addition, the variegated testa represents a distinct regulation pattern of anthocyanin accumulation in integument cells.

**Methods:**

In order to identify the genetic locus underlying variegated testa color in peanut, two populations were constructed from the crosses between Fuhua 8 (pure-pink testa) and Wucai (red on white variegated testa), Quanhonghua 1 (pure-red testa) and Wucai, respectively. Genetic analysis and bulked sergeant analysis sequencing were applied to detect and identify the genetic locus for variegated testa color. Marker-assisted selection was used to develop new variegated testa peanut lines.

**Results:**

As a result, all the seeds harvested from the F1 individuals of both populations showed the variegated testa type with white trace. Genetic analysis revealed that the pigmentation of colored region in red on white variegated testa was controlled by a previous reported gene AhRt1, while the formation of white region (un-pigmented region) in variegated testa was controlled by another single genetic locus. This locus, named as AhVt1 (Arachis hypogaea Variegated Testa 1), was preliminary mapped on chromosome 08 through bulked sergeant analysis sequencing. Using a secondary mapping population derived from the cross between Fuhua 8 and Wucai, AhVt1 was further mapped to a 1.89-Mb genomic interval by linkage analysis, and several potential genes associated with the uneven distribution of anthocyanin, such as MADS-box, MYB, and Chalcone synthase-like protein, were harbored in the region. Moreover, the molecular markers closely linked to the AhVt1 were developed, and the new variegated testa peanut lines were obtained with the help of marker-assisted selection.

**Conclusion:**

Our findings will accelerate the breeding program for developing new peanut varieties with “colorful” testa colors and laid a foundation for map-based cloning of gene responsible for variegated testa.

## 1 Introduction

Variegation is an interesting phenomenon widely observed in many plant species (Marcotrigiano, 1997). It develops differently colored sectors in the surface of plant organs, such as leaves, stems, flowers, fruits, and seeds. In natural condition, the variegated leaves may help plants reduce the harm from the herbivore (Campitelli et al., 2008), and the variegated flowers may affect pollinator preferences (Davies et al., 2012; Glover et al., 2013). In the market, plants with attractive variegation in leaves, flowers, and fruits are preferred by the consumers and usually have higher commercial value (Behe et al., 1999; Chen et al., 2014). Hence, it is meaningful for us to uncover the mystery underlying variegation in plants.

It is believed that an uneven distribution of pigments in certain cells of variegated region is the main physiological reason for variegation (Wu et al., 2017). There are three major classes of plant pigments: anthocyanin, chlorophyll, and carotenoid. Of them, anthocyanin is the main pigment in flowers, fruits, and seeds and is responsible for red, violet, and blue colors, while carotenoid widely spread in various organs paints the plants with bright red, orange, and yellow colors, and chlorophyll is the fundamental pigment in leaves and confers green color (Tanaka et al., 2008).

Until now, most of the studies in plants revealed that the distinct expression patterns of anthocyanin biosynthesis structure genes or regulatory genes led to the un-balanced accumulation of anthocyanin, which were the main reasons for the formation of variegation. In some cases, the transposon elements influenced the anthocyanin accumulation by inserting or excising the anthocyanin biosynthesis genes or regulatory genes (Schiefelbein et al., 1988). In some other cases, exogenous RNA interference (RNAi) induced *via* virus or DNA methylation inhibited the expression of anthocyanin biosynthesis genes or regulatory genes in certain cells, leading to variegation (Nishizaki et al., 2011; Jiang et al., 2020). Studies also revealed that the specific transcriptional factors were involved in variegation formation. For example, the spatially and temporally distinct expression of MYB transcriptional factors resulted in the development of variegated color patterns in the petal of phalaenopsis (Hsu et al., 2015), tree peony (Gu et al., 2019), the peel of apple (Telias et al., 2011), the tepal of hybrid lilies (Suzuki et al., 2016), and so on. The MADS-box transcriptional factors were also considered as important regulatory factors for the bicolor pattern in ray florets (Qi et al., 2022), cattleya hybrida (Li et al., 2020). Furthermore, recent researches indicated that miRNA played an important role in the unstable distribution of pigments in cells (Tirumalai et al., 2019; Yamagishi and Sakai, 2020). All these results suggested the complexity of molecular mechanisms underlying variegation formation.

Peanut (*Arachis hypogaea* L.) is an important oil and economic crop in the world. Unlike most of the peanut varieties that show pure-pink testa color, a unique series of peanut varieties exhibit red on white variegated testa color. The peanut seeds with this variegated testa color have attractive appearance and a higher market value than ordinary peanut seeds. In addition, some studies claimed that this phenotype may be associated with other important agronomic traits, such as sugar content and nodulation habit (Dashiell et al., 2001; Hu et al., 2021). Therefore, it is important to dissect the molecular mechanism underlying the formation of variegated testa color in peanut. It is known that the major pigment in peanut seed testa is anthocyanin (Xue et al., 2021). A study compared transcripts and metabolites in the red-zone and white-zone of the same developing variegated testa, using transcriptomic and metabolomics joint analysis. It showed that the differently expressed genes (DEGs) were enriched in the anthocyanin biosynthesis and accumulation pathways, suggesting that cyanidin and delphinidin were the primary metabolites that caused the uneven distribution of pigments in two regions (Hu et al., 2021). A previous study revealed that the red on white variegated testa color was controlled by one single gene (the *v* gene) in peanut (Branch and Hammons, 1979). Although some progress have been made, the position for genetic locus and gene responsible for the red on white variegated testa color is still obscure to us, limiting the process of uncovering the mechanism underlying the phenotype and the breeding program for new “colorful” peanut varieties.

Bulked-segregant analysis is a strategy of genetic mapping by constructing two bulks of individuals with contrasting phenotypes from the segregating population. Bulked-segregant analysis sequencing (BSA-seq), based on next-generation sequencing technology, is an effective tool in mapping genetic loci underlying agronomic traits (especially for those quantitative traits) (Li and Xu, 2022). In peanut, this method has been applied for mapping genetic loci responsible for agronomic traits, such as late leaf spot resistance (Clevenger et al., 2018; Han et al., 2022), seed dormancy (Kumar et al., 2020), seed sucrose content (Guo et al., 2022), growth habit (Li et al., 2022; Pan et al., 2022), and testa color (Chen et al., 2021; Zhang et al., 2022).

In this study, to understand the inheritance of variegated testa in peanut, genetic analysis was performed in the segregating populations, using a variegated testa peanut landrace as a parent line. It revealed that the variegation phenotype was controlled *via* single genetic locus. This locus was mapped in chromosome 08 by BSA-seq. New variegated testa peanut lines have also been produced with the help of newly developed molecular markers.

## 2 Materials and methods

### 2.1 Plant material

“Wucai (Wc)” is a peanut landrace with red on white variegated testa color collected from Shandong Province, China. “Fuhua 8 (FH8)” is a peanut cultivar with pink testa color (containing *AhRt1_P_* allele) cultivated by Fujian Academy of Agricultural Sciences, China. Using FH8 as female parent, and Wc as male parent, FH8 was crossed with Wc to generate F_1_ plants, and the F_2_ population was constructed by selfing the F_1_ plants. The F_2_ population derived from the cross between FH8 and Wc was used for genetic analysis and BSA-seq. The advanced segregating population of this cross was used for further mapping analysis by selfing the F_2_ plants heterozygous in the targeting region. “Quanhonghua 1 (QH1)” is a red testa color peanut cultivar containing *AhRt1_R_* allele developed by Quanzhou Institute of Agricultural Sciences, China. Another F_2_ population was constructed to conduct progeny test, using QH1 as female parent, and Wc as male parent. “Yunnan Black (YB)” is a peanut landrace collected from Yunnan Province, China. It contains *AhTc1_B_* allele responsible for dark purple/black testa color, and was used as the donor for purple/black testa color for new variegated lines.

### 2.2 Quantification of anthocyanin in peanut testa

The anthocyanin content in testa was measured based on the UV visible spectroscopy method described in the previous study (Giusti and Wrolstad, 2001).

### 2.3 Determination of testa color type

The color type of testa was determined by the area of unpigmented region (white region), using visual observation. The unpigmented region of pink on white variegated testa (PW) individuals and red on white variegated testa (RW) individuals take up more than half of the surface in testa, while the unpigmented region in red with white trace (RWT) individuals and pink with white trace (PWT) individuals just take up less than a quarter of the surface in testa. The difference of color type in testa can be examined by the unaided eye.

### 2.4 Prepare for DNA bulks and illumina sequencing

We collected the young leaves of the parent line Wc, FH8, 10 individuals showing pure pink testa (PP), 20 individuals showing pure red testa (PR), 10 individuals showing pink on white variegated testa (PW), and 20 individuals showing red on white variegated testa (RW) from the F_2_ population derived from the cross between FH8 and Wc.

The collected young leaves were used for extracting DNA, respectively, following a modified CTAB method. We evaluated the quality and concentration of the DNA for each sample by 1% agarose gel electrophoresis and NanoDrop. The Pure Testa Bulk (PTB) pool was generated by mixing the equal amount of total DNA for each collected PP and PR DNA samples, and the Variegated Testa Bulk (VTB) pool was generated by mixing the equal amount of total DNA for each collected PW and RW DNA samples. The DNA libraries (including PTB, VTB, and the parent line Wc, FH8) were constructed by following the protocol of the NEB Next Ultra II DNA Library Prep Kit for Illumina, and the high-throughput sequencing of DNA libraries was performed by Illumina NovaSeq platform with NovaSeq 6000 S4 Reagent Kit in Genoseq Technology Co. Ltd (Wuhan, China). The raw data of Illumina sequencing data have been deposited in GenBank (BioProject: PRJNA681634 and PRJNA925001).

### 2.5 Variant detection and BSA-seq

The high-quality clean data were obtained from the Illumina sequencing data, after removing the low-quality reads (Q <20), adapter sequences, N > 10% reads, and too short reads (<50 bp) by the software of fastap. We aligned the obtained high-quality clean data to the reference cultivated peanut genome (Tifrunner, https://peanutbase.org/data/v2/Arachis/hypogaea/genomes/Tifrunner.gnm2.J5K5/), using the BWA software (Li and Durbin, 2009). The process of variant detection and annotation was described in a previous study (Chen et al., 2021). In general, after getting the alignment result by BWA, we used the SAMTOOLS to remove the duplicates (Li et al., 2009), and used “SortSam” in “Picard” tools to remove the PCR repeats. The variation detection was conducted by using the “HaplotypeCaller” module of “GATK” software (Magwene et al., 2011), and the annotation of variant was carried out by ANNOVAR (Wang et al., 2010).

We performed the BSA-seq *via* QTLseqr R package (Mansfeld and Grumet, 2018). The variants from the PTB and VTB were selected according to a previous study (Chen et al., 2021). The determination of the genomic region responsible for variegated testa was based on Δ (SNP-index) of genomic regions from PTB and VTB DNA pools (Takagi et al., 2013). In this study, the genomic region with the Δ (SNP-index) value higher than threshold calculated with 10000 permutations under p < 0.01 level was considered as the candidate region with gene controlling variegated testa color.

### 2.6 Genetic mapping using the flanking marker method

A segregation population containing 1006 individuals was generated from a cross between FH8 and Wc. The plants from this segregating population were used for selecting the recombination in the genomic region flanking *AhVt1*. Two markers, PA01 and P10, which flanked *AhVt1*, were firstly used to detect the recombination events that occurred in the preliminary mapping region. Nine new SNP markers (PA2, PA4, PA5, PA6, PA7, PA8, PB7, PC4, and PC6) distributed in the *AhVt1* mapping region were developed. The order of the markers and the genetic distance between every two adjacent markers in the target region were determined by linkage analysis. All the recombinants were genotyped and the phenotype for testa color was evaluated. The recombination condition of the target gene and these markers was also analyzed. Finally, the mapping interval of *AhVt1* was determined by flanking marker method.

### 2.7 Development of molecular markers

A total of 11 new SNP markers (PA01, PA2, PA4, PA5, PA6, PA7, PA8, PA10, PB7, PC4, and PC6) were further developed based on the re-sequencing result of two parent lines (FH8 and Wc). In addition, the markers PF1, PF2, and PF3 used for marker-assisted selection were developed based on the information from this study and previous studies *(* Chen et al., 2021 *;* Huang et al., 2020 *;* Zhao et al., 2020). All these SNP markers were developed by the procedure described in the previous study (Campbell et al., 2015). The further sequence information of all primers used in this study is listed in **Supplementary Table S1**.

### 2.8 Gene annotation in the mapping interval of *AhVt1*

According to the physical position for the mapping region of *AhVt1*, the annotated information of putative genes in the mapping region was obtained from Peanutbase (https://peanutbase.org/data/v2/Arachis/hypogaea/annotations/Tifrunner.gnm2.ann1.4K0L/). The DEGs profile between the red pigmented region and white non-pigmented region of developing testa was obtained from a previous study (Hu et al., 2021). Fold change ≥2 and FDR <0.01 were considered as the screening criteria for DEGs.

## 3 Result

### 3.1 Genetic analysis of the variegated testa color in peanut

**Figure 1:**
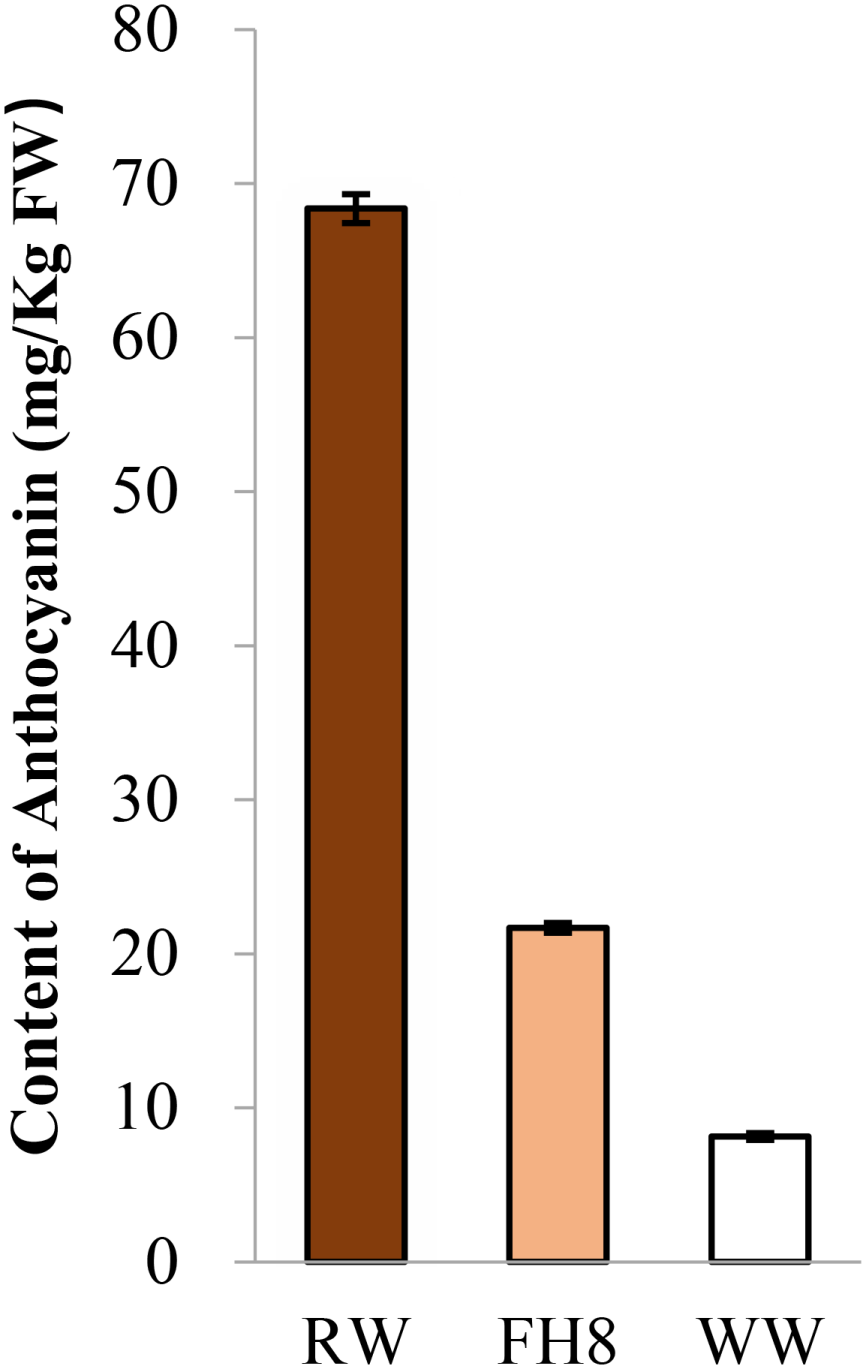
Comparison of the anthocyanin content in testa from different testa pigmentation pattern peanut germplasm. Red zone on Wc (RW), white zone on Wc (WW), and pink zone on Fuhua 8.

**Figure 2:**
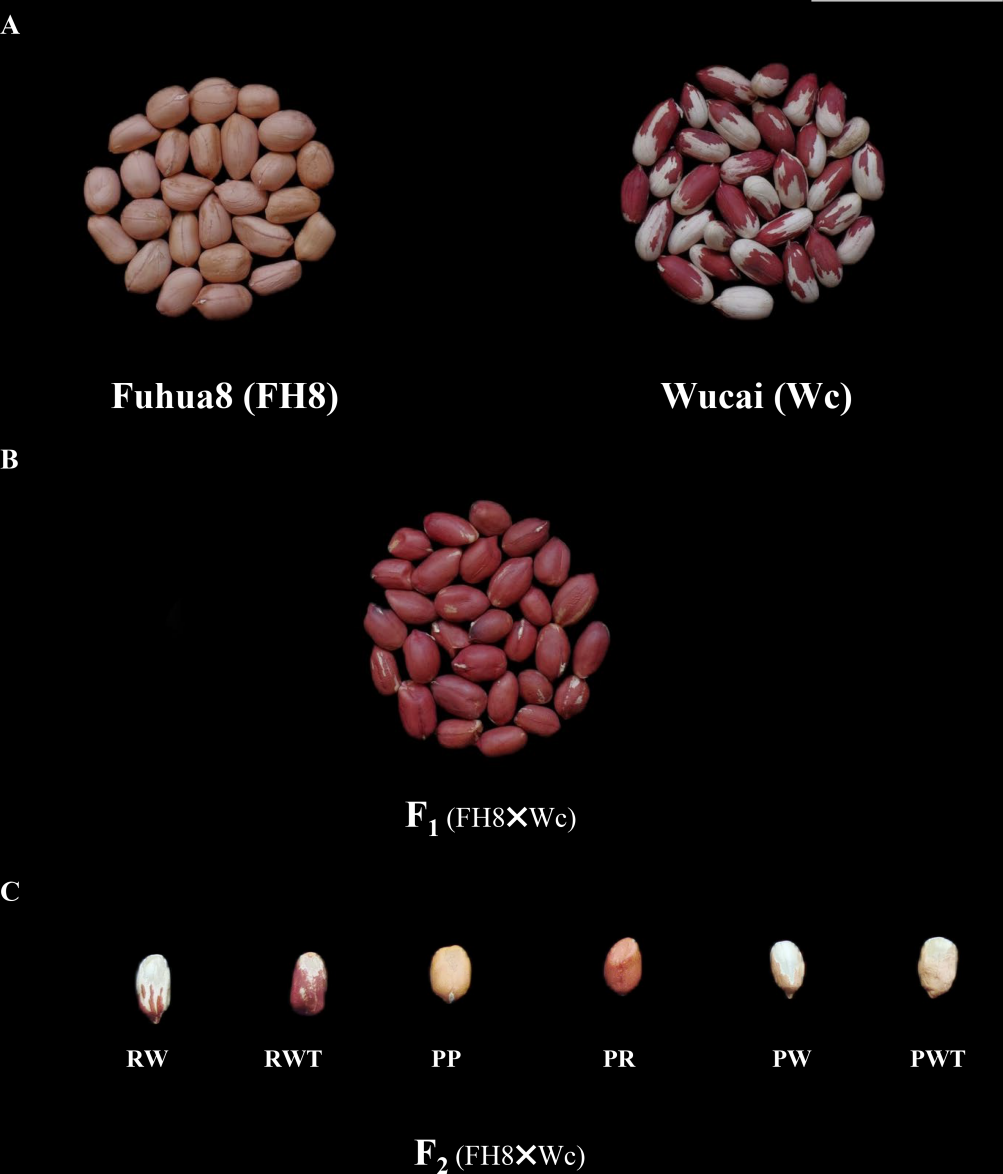
Comparison of testa color of peanut cultivar “Fuhua 8” and “Wucai”, and the genetic analysis for variegated testa color in peanut. **(A)**. Seeds of pink testa cultivar “Fuhua 8” and red on white variegated testa color landrace “Wucai”. Scale bar = 5 cm. **(B)**. The red on white trace testa seeds harvested from F_1_ plants derived from the cross between “Fuhua 8” and “Wucai”. **(C)**. The segregation of testa pigmentation pattern of seeds harvested from F_2_ individuals derived from the cross between “Fuhua 8” and “Wucai”. Pure red (PR), pure pink (PP), red on white variegated (RW), pink on white variegated (PW), red with white trace (RWT), and pink with white trace (PWT).

The peanut landrace Wucai (Wc) showed the red on white variegated testa color. It is known that the major pigment in peanut testa is anthocyanin. Hence, we measured the anthocyanin content in the different pigmented zones of Wc testa, while the testa from a pure pink peanut cultivar Fuhua 8 (FH8) was also measured as control. As expected, the red zone in Wc exhibited the highest level of anthocyanin content, while the white unpigmented zone displayed the lowest anthocyanin content (**Figure 1**). In order to identify the gene controlling the red on white variegated testa color in peanut, a cross between FH8 with pure-pink testa and Wc with red on white variegated testa color was conducted (**Figure 2A**). All the seeds harvested from F_1_ individuals showed the same “red with white trace” variegated pigmentation pattern in testa. However, compared with their red on white variegated testa color parent Wc, the “red with white trace” variegated testa exhibited an obvious increase in the area with red pigmentation and a decrease in the white un-pigmented area (**Figure 2B**). We further investigated the testa color in the F_2_ population, and found that the testa color of the seeds from F_2_ individuals could be segregated into six types: pure red (PR), pure pink (PP), red on white variegated (RW), pink on white variegated (PW), red with white trace (RWT), and pink with white trace (PWT) (**Figure 2C**), with an approximate segregation ratio of 3: 1: 3: 1: 6: 2, respectively (χ^2^ = 1.316<11.070 at degree of freedom, *df* = 5, and p = 0.05). Interestingly, the number of red testa individuals (including PR + RW + RWT) and the pink testa individuals (including PP + PW + PWT) in the F_2_ population was consistent with a 3:1 ratio (χ^2^ = 0.034<3.841 at df = 1, and p = 0.05), while the number of pure-color testa individuals (including PR+PP), the variegated testa with white trace individuals (including RWT+PWT), and the “pure” variegated testa individual (including PW+RW) in the F_2_ population were consistent with a 1: 2: 1 ratio (χ^2^ = 0.944<5.991 at df = 2, and p = 0.05) (**Table 1**). These results suggested that the red/pink color and variegation in the testa were controlled by two independently inherited genes, respectively. The red/pink color in testa was controlled by a dominant gene, while the variegated pigmentation in testa was controlled by a co-dominant gene.

**Table 1:** Genetic analysis of variegated testa color.

Population	Segregation condition for testa color	Proposal model	Chi-square test
F_1_ (FH8×Wc)	RWT (15)		
F_2_ (FH8×Wc)	PR (25), PP (9), RW (30), PW (11), RWT (63), PWT (18)	3: 1: 3: 1: 6: 2	χ^2^ = 1.316<11.070 (*df* = 5, p = 0.05)
	Variegated Pattern: PV (41), VT (81), PC (34)	1: 2: 1	χ^2^ = 0.944<3.841 (*df* = 2, p = 0.05)
	Color Pattern: Red (118), Pink (38)	3: 1	χ^2^ = 0.034<5.991 (*df* = 1, p = 0.05)
F_1_ (QH1×Wc)	RWT (11)		
F_2_ (QH1×Wc)	Variegated Pattern: PV (31), VT (78), PC (36)	1: 2: 1	χ^2^ = 1.179<5.991 (*df* = 2, p = 0.05)
	Color Pattern: Red (145)		

The number in the parentheses represents the number of individuals, pure red (PR), pure pink (PP), red on white variegated (RW), pink on white variegated (PW), red with white trace (RWT), and pink with white trace (PWT) represent different testa coloration type in population, degree of freedom (df).

As our previous study has reported a dominant locus *AhRt1* conferring the red testa color in peanut (Chen et al., 2021), we hypothesized that the red color in this study was also controlled *via AhRt1*. A marker PF3 tightly linked to *AhRt1* was used to confirm this hypothesis in the F_2_ population. It was shown that the marker can classify the red and pink individuals in F_2_ population. We further constructed another F_2_ population derived from a cross between Quanhonghua 1 (QH1, pure red testa, and containing *AhRt1_R_*) and Wc. The testa color of the seeds from the plants in this F_2_ population was just segregated into three types: pure red (PR), red with white trace (RWT), and red on white variegated (RW), with a segregation ratio of 1: 2: 1(χ^2^ = 1.179<5.991 at df = 2, and p = 0.05) (**Table 1**). The color in the testa was not segregated in this F_2_ population, while the variegation type in testa was segregated.

Taken all of these results together, it was indicated that the red color in the red on white variegated testa was controlled by *AhRt1*; the variegation in testa was actually controlled by one single gene. We named it as *AhVt1* (*Arachis hypogaea Variegated Testa 1*).

### 3.2 BSA-seq identified *AhVt1*

**Figure 3:**
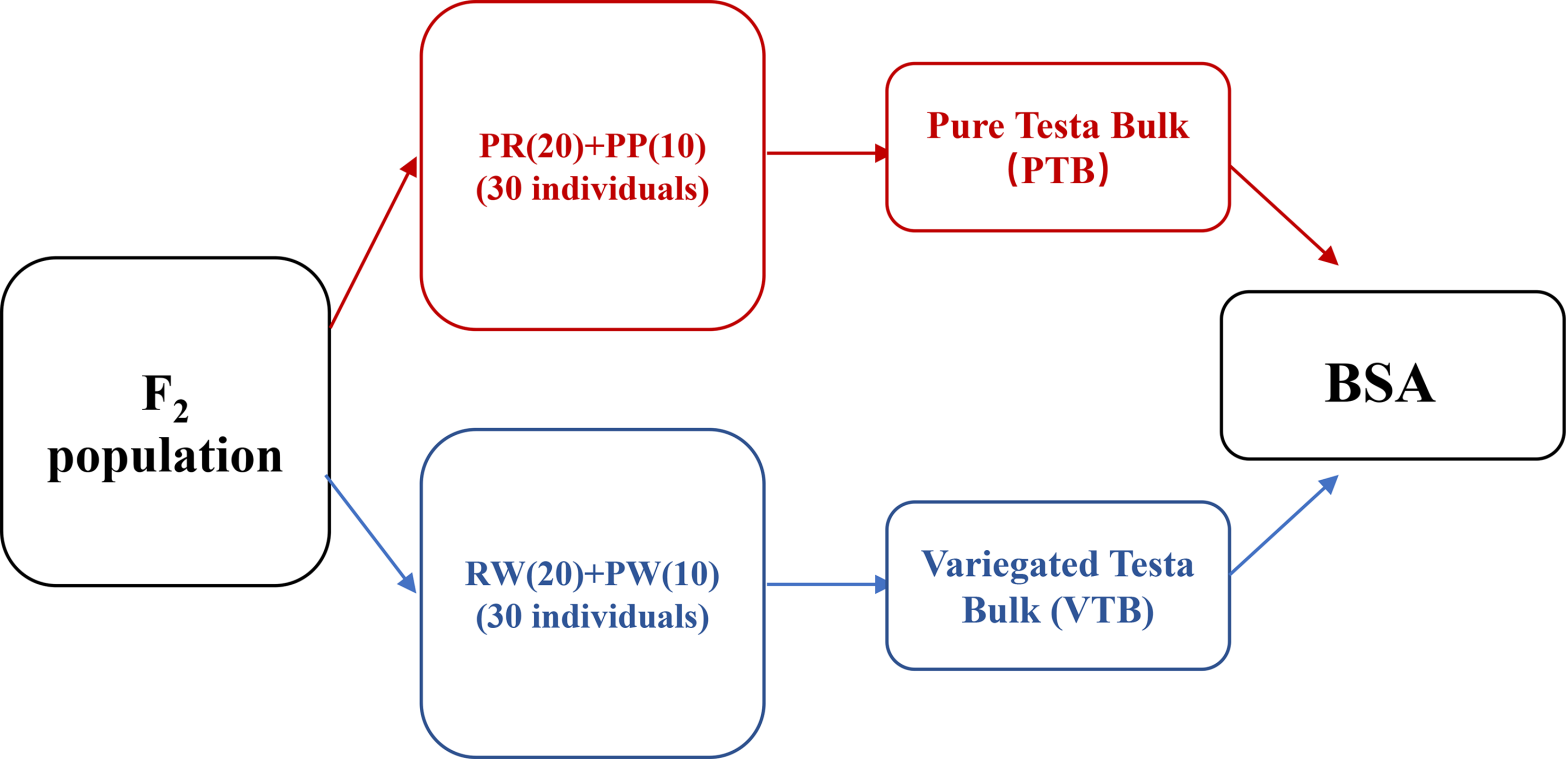
A simplified overview of our BSA-seq.

**Figure 4:**
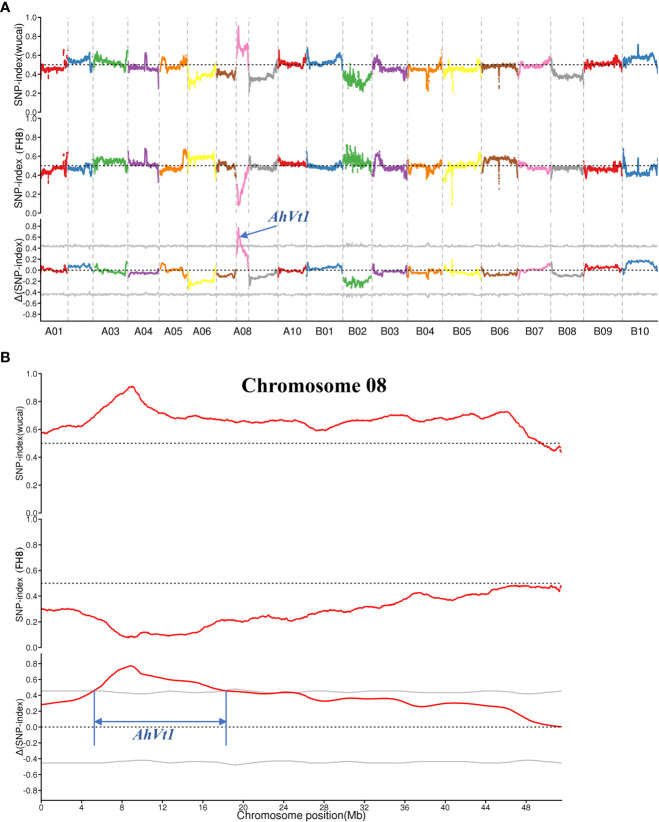
Mapping of locus responsible for variegated testa by BSA-seq strategy. **(A)**. SNP and ΔSNP index of the Pure Testa Bulk (PTB) and Variegated Testa Bulk (VTB) in the whole genome-wide scale. **(B)**. Preliminary mapping of *AhVt1* in chromosome 08. The gray lines indicate the threshold value. The preliminary mapping region of *AhVt1* was limited by two blue lines.

The BSA-seq was conducted to map *AhVt1*. We randomly selected 30 individuals with “pure” testa color (including PR and PP) and 30 individuals with variegated color testa (including RW and PW) in the F_2_ population derived from the cross between FH8 and Wc. The DNA from selected F_2_ individuals was pooled together to construct two DNA bulks: Pure Testa Bulk (PTB) and Variegated Testa Bulk (VTB), respectively, based on their testa color (**Figure 3**). We re-sequenced the PTB, VTB, and the parent lines Wc and FH8 by the whole-genome re-sequencing method. The whole-genome re-sequencing generated 261,215,250 reads from PTB (average 25.0× depth and 94.83% coverage, using the Tifrunner as reference genome), 286,939,221 short reads from VTB (27.34× depth and 94.88% coverage), 172,382,458 short reads from wucai (16.59× depth and 93.57% coverage), and 79,241,708 short reads from FH8 (8.67× depth and 90.27% coverage) (**Table 2**). A total of 322,377 SNP and 37,355 Indel markers uniformly and evenly distributed in the genome of cultivated peanut have been identified by comparing the re-sequencing data from PTB, VTB, Wc, and FH8 (**Supplementary Table S2**). Using the SNP and Indel variants data from PTB and VTB, the SNP-index and Δ (SNP-index) for two bulks were analyzed and plotted against the position of the reference genome (**Figure 4A**). Most of the genomic regions showed no significant difference in SNP-index for PTB and VTB, demonstrating that they were not relevant to the variegated color testa phenotype. Interestingly, one unique genomic region comprised 6.21 to 19.17 Mb on chromosome 08 exhibited unequal contributions from two parent lines, and the Δ (SNP-index) value was significantly higher than the cut off value, indicating that this region was highly relevant to the variegated color testa (**Figure 4B**). It also conformed to the result of genetic analysis. Hence, we regarded this genomic region as the preliminary mapping region for *AhVt1*.

**Table 2:** Summary of sequencing data.

Sample	Reads	Q30(%)	Unique-mapped (%)	Repeatedly-Mapped (%)	Coverage (%)	Depth
FH8	79,241,708	96.20	86.00	12.98	91.48	8.67
Wc	172,382,458	93.22	81.08	13.08	90.12	16.59
PTB	261,215,250	94.90	81.39	14.23	94.87	25.00
VTB	286,939,221	94.44	80.97	14.39	94.97	27.34

Q30 is short for quality score. The quality score is logarithmically linked to error probability.

### 3.3 Narrowing down the mapping region of *AhVt1*

**Figure 5:**
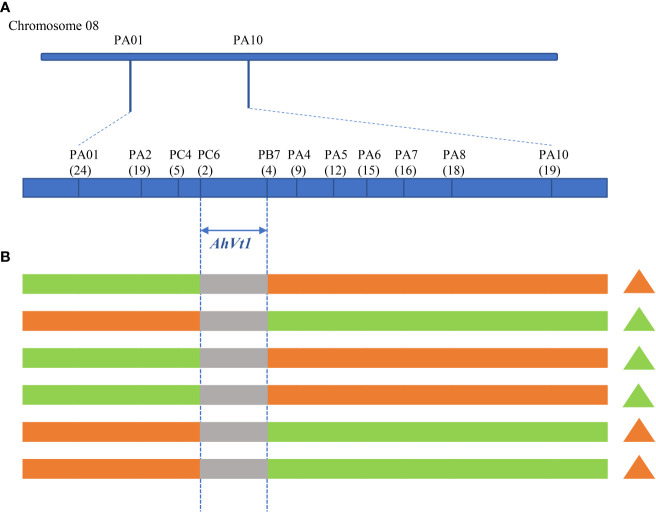
Narrow down the mapping region of *AhVt1.* **(A)**. Genetic mapping of *AhVt1* on chromosome 08 based on individuals from the segregating population. The number of recombinants between adjacent markers is indicated under the linkage map. **(B)**. The genotype of important recombinants that determine the mapping region of *AhVt1*. The green bar represents the genomic segment from FH8, the orange bar represents the genomic segment from Wc, the grey bar represents the genomic segment where the recombination occurs. The triangle represents the testa phenotype of corresponding recombinant. The green triangle represents the pure-color testa, while the orange triangle represents the variegated testa color.

To reduce the mapping interval of *AhVt1*, we generated a segregating population containing 1006 individuals derived from the cross between FH8 and Wc. Two markers, PA01 and PA10, which flanked the *AhVt1* preliminary mapping region, were developed to screen the recombinant individuals. A total of 43 recombinants between PA01 and PA10 were identified. Nine markers (PA2, PC4, PC6, PB7, PA4, PA5, PA6, PA7, and PA8) distributed across the mapping region were further developed to genotype these recombinants. A high-resolution map flanking the *AhVt1* mapping region was constructed. We evaluated the genotype and phenotype of the recombinant individuals. *AhVt1* was narrowed down to a 1.89-Mb genomic region between markers PC6 and PB7 (**Figure 5**). A total of 93 predicted genes were presented in this region, based on a cultivated peanut sequence annotation database (**Supplementary Table S3**).

To explore the nucleotide variants in *AhVt1* mapping region, the re-sequencing data of two parent lines, FH8 and Wc, were compared and analyzed. As a result, a total of 82 SNPs and 15 Indels were detected in the genome of the *AhVt1* mapping region. We further annotated these SNPs/Indels, and there were two SNPs located on the exon, three SNPs and two Indels located on the intronic region, three SNPs and two Indels located on the 5′UTR or 3′UTR region, five SNPs located on the upstream/downstream of genes, and one SNP located on the splicing junction of intronic/exonic. The remaining SNPs and Indels were harbored in the intergenic region (**Supplementary Table S4**).

The putative differentially expressed genes (DEGs) in the *AhVt1* mapping region were predicted by comparing the published transcriptomic data between the pigmented (red) and unpigmented areas (white) in the developing red on white variegated testa (Hu et al., 2021). A total of four DEGs were identified in the *AhVt1* mapping region, including the *arahy.NKL1UU* encoding MADS-box transcription factor family protein, *arahy.33MSFM* encoding senescence-inducible chloroplast stay-green protein 2, *arahy.AG2MXP* encoding transmembrane amino acid transporter family protein, and *arahy.FQ5ZUR* encoding response regulator. Among them, *arahy.NKL1UU*, *arahy.33MSFM* and *arahy.FQ5ZUR* showed significant higher expression level in the unpigmented zone than that in the red zone, while *arahy.AG2MXP* exhibited the opposite expression tendency (**Supplementary Table S5**).

### 3.4 Developing new variegation testa pattern peanut lines using the marker-assist selection approach

**Figure 6:**
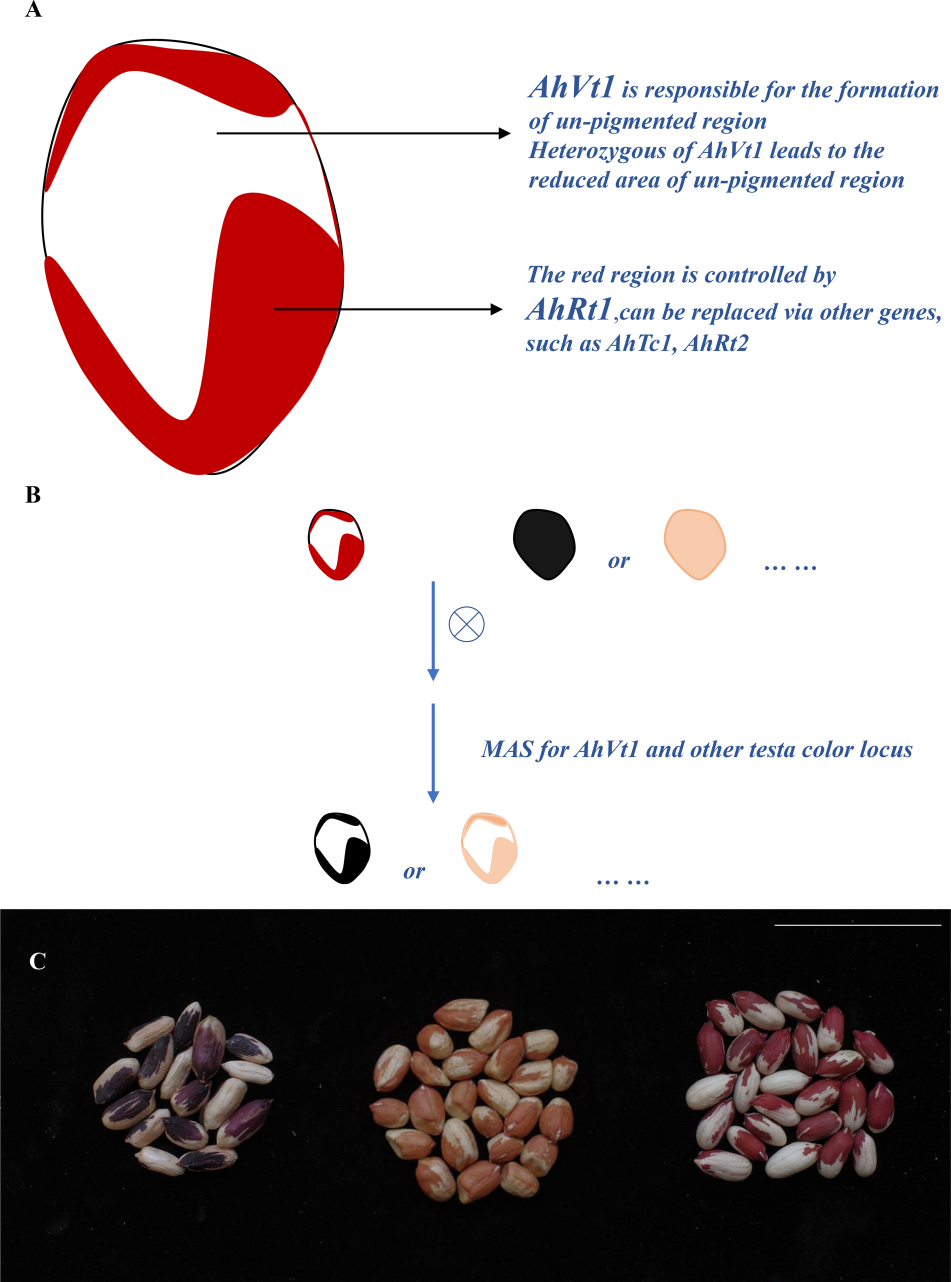
Development new variegation testa pattern peanut lines by marker-assist selection (MAS) approach. **(A)**. Work model for *AhVt1* in formatting the variegation testa. **(B)**. Development of the new variegation testa pattern peanut lines by MAS. **(C)**. The seed of new developed black on white variegated and pink on white variegated testa peanut lines. Scale bar = 5 cm.

As the genetic basis of variegation in testa pigmentation of peanut has been uncovered (**Figure 6A**), new peanut lines with variegated testa can be developed by directional selection of two loci (one variegated testa locus and one testa color locus), using the MAS system. For example, we developed a new black on white variegated testa line by conducting a cross between a peanut landrace “Yunnan Black (YB)” with black/deep purple testa (conferred *via AhTc1_B_*) and Wc with the red on white variegated testa. As the black testa in YB was controlled *via* a previous reported allele *AhTc1_B_*, we used a marker PF2 tightly linked to *AhTc1*, and a marker PF1 tightly linked to *AhVt1* to screen the F_2_ and F_3_ population derived from the cross between YB and Wc (**Figure 6B**). As a result, a stable black on white variegated testa peanut line was rapidly obtained by MAS (with the combination of alleles *AhTc1_B_AhTc1_B_/AhVt1_V_AhVt1_V_* (**Figure 6C**). We can also obtain the causal individuals with black with white trace testa, purple with white trace testa, and purple on white variegated testa by MAS, if necessary. In addition, the new type pink on the white variegated testa line (with the combination of alleles *AhRt1_P_AhRt1_P_/AhVt1_V_AhVt1_V_*) was also obtained by screening the population derived from FH8 and Wc with the similar procedure by the markers PF1 and PF3 (**Figure 6C**).

## 4 Discussion

Anthocyanin is known to be the major pigment of testa in peanut. The content and composition of anthocyanin co-determine the color of peanut testa, and also give different types of peanut testa distinct nutritional and healthcare functions (Zhang et al., 2022). Understanding the regulation mechanisms for the accumulation of anthocyanin in integument also uncovers the regulation of pigmentation in peanut testa. People have systematically studied the mechanism of peanut testa color for over 100 years (Branch, 2011). Especially in the recent years, with the rapid progress in peanut functional genomics research (Chen et al., 2016; Yin et al., 2018; Bertioli et al., 2019; Zhuang et al., 2019), many genetic loci underlying the peanut testa color have been identified, such as *AhTc1* for black/purple color (Huang et al., 2020; Zhao et al., 2020) and *AhRt1* and *AhRt2* for red color (Chen et al., 2021; Zhang et al., 2022), respectively. These studies laid the foundation for uncovering the genetic mechanisms of peanut testa color formation and breeding for new peanut varieties with different colors in testa. In this study, we identified a new genetic locus responsible for variegated testa color in chromosome 08.

According to our result, we conclude that this red on white variegated testa phenotype is controlled *via* two types of loci. One type of locus is *AhVt1*; this type of locus is responsible for the formation of variegation phenotype. The allele from Wc (*AhVt1_V_*) leads to a lack of pigmentation ability for part of the epidermal cells in seed coat, therefore exhibiting white color in seed coat. The heterozygous of *AhVt1_V_* allele also leads to a lack of pigmentation ability for some of the epidermal cells in seed coat, but non-pigmented region for heterozygous *AhVt1_V_* genotype is smaller (fewer non-pigmented cells) compared to that in the homozygous *AhVt1v* allele, while the homozygous *AhVt1_P_* allele led to a pure color testa without any non-pigmented region. This suggests a semi-dominant effect of *AhVt1_V_*, when compared to *AhVt1_P_*. The other type of locus is the normal testa color genes that control pigmentation of the remaining of the testa epidermal cells in variegated seed coat. This gene could be *AhRt1* underlying red testa color as in Wc, and it could be replaced *via* other seed coat genes, such as *AhTc1*, *AhRt1*, or other testa color genes in peanut. Hence, we can directly and effectively generate new type variegated testa peanut lines *via* hybridization and MAS. In fact, we have obtained a stable-inherited black on white variegated testa line by MAS in two generations.

*AhVt1* has been delimited to a 1.89-Mb genomic region, and there were 93 genes in this genomic region. Of these, *arahy.H1H5BB*, *arahy.NKL1UU*, *arahy.M75BKX*, and *arahy.33MSFM* are more interesting candidate genes for *AhVt1*. *arahy.H1H5BB*, as well as *arahy.LY4YXE* and *arahy.FM6CZ7* in the mapping region, which encode a Chalcone synthase (CHS)-like protein. CHS is known to be a key enzyme to catalyze the first reaction which produces cyanidin 3-O-ß-rutinoside in the biosynthesis pathway of flavonoids. The expression level and spatial expression pattern of CHS genes has been reported to be associated with the accumulation of anthocyanin in different tissues, and to finally influence the pigmentation of tissues in many species (Morita et al., 2012; Deng et al., 2014). In addition, one annotated SNP was located on the upstream of *arahy.H1H5BB*. *arahy. NKL1UU* belongs to MADS-box transcription factor family protein. MADS-box transcription factors can regulate anthocyanin biosynthesis by influencing MYB gene transcription (Jaakola et al., 2010), or though regulating the expression of anthocyanin biosynthesis structure genes (Wu et al., 2014). Nevertheless, the MADS-box transcription factors can also influence bicolor pattern formation in cineraria ray florets (Qi et al., 2022). The predicted gene *arahy.M75BKX* encodes the MYB transcription factor, and recent studies have reported that MYB genes have participated in the differential coloration formation (Hsu et al., 2015; Gu et al., 2019). *arahy.33MSFM* encoding senescence-inducible chloroplast stay-green protein 2 (SGR2) were also harbored in the mapping region. *SGR2* is a negative regulator of chlorophyll degradation during leaf senescence, and it determines the seed coat color in pea (Armstead et al., 2007). Among four DEGs identified in the mapping region, *arahy.NKL1UU* showed a significantly higher expression level in the un-colored zone than that in the red zone of testa. It coincided with the expressional tendency of the MADS-box transcriptional factor *ScAG* and *ScAGL11* in cineraria ray florets. *ScAG* and *ScAGL11* negatively regulated the anthocyanin biosynthesis and influenced the bicolor pattern formation (Qi et al., 2022). Therefore, *arahy.NKL1UU* may have the similar function in variegation formation. However, the candidate genes should be validated by the future research.

In conclusion, identification of *AhVt1* helps us uncover the genetic rule for variegated testa color in peanut, and improves the breeding programs of new “colorful” peanut varieties.

## Data availability statement

The datasets presented in this study can be found in online repositories. The names of the repository/repositories and accession number(s) can be found below: https://www.ncbi.nlm.nih.gov/genbank/, PRJNA681634, https://www.ncbi.nlm.nih.gov/genbank/, PRJNA925001.

## Author contributions

HC and HJ conceived and designed the research. HC, RX, XC, HZ, YC, and JC developed the populations, planted the materials, and conducted field management. WL performed the measurement of anthocyanin. HC, XY, NL, LH, and HL performed the BSA-seq, mapping, and gene candidate analysis. HC, LH, and XY developed the molecular markers and new variegated lines. HC wrote the manuscript; DH, HL, XY, and HJ revised the manuscript. All authors contributed to the article and approved the submitted version.
